# Impact of “hypotension on arrival” on required surgical disciplines and usage of damage control protocols in severely injured patients

**DOI:** 10.1186/s13049-024-01187-0

**Published:** 2024-05-14

**Authors:** Moritz Witzenhausen, Björn Hossfeld, Martin Kulla, Christian Beltzer

**Affiliations:** 1 Department of General, Abdominal and Thoracic Surgery, German Armed Forces Hospital Ulm, Oberer Eselsberg 40, 89081 Ulm, Germany; 2 Department of Anaesthesiology, Intensive Care Medicine, Emergency Medicine and Pain Therapy, German Armed Forces Hospital Ulm, Oberer Eselsberg 40, 89081 Ulm, Germany; 3 Bundeswehrkrankenhaus Ulm, Ulm, Germany

**Keywords:** Trauma surgery, Hypotension on arrival, Severe trauma, Damage control protocol

## Abstract

**Background:**

For trauma patients with subsequent immediate surgery, it is unclear which surgical disciplines are most commonly required for treatment, and whether and to what extend this might depend on or change with “hypotension on arrival”. It is also not known how frequently damage control protocols are used in daily practice and whether this might also be related to “hypotension on arrival”.

**Methods:**

A retrospective analysis of trauma patients from a German level 1 trauma centre and subsequent “immediate surgery” between 01/2017 and 09/2022 was performed. Patients with systolic blood pressure > 90 mmHg (group 1, no-shock) and < 90 mmHg (group 2, shock) on arrival were compared with regard to (a) most frequently required surgical disciplines, (b) usage of damage control protocols, and (c) outcome. A descriptive analysis was performed, and Fisher’s exact test and the Mann‒Whitney U test were used to calculate differences between groups where appropriate.

**Results:**

In total, 98 trauma patients with “immediate surgery” were included in our study. Of these, 61 (62%; group 1) were normotensive, and 37 (38%, group 2) were hypotensive on arrival. Hypotension on arrival was associated with a significant increase in the need for abdominal surgery procedures (group 1: 37.1 vs. group 2: 54.5%; *p* = 0.009), more frequent usage of damage control protocols (group 1: 59.0 vs. group 2: 75.6%; *p* = 0.019) and higher mortality (group 1: 5.5 vs. group 2: 24.3%; *p* 0.027).

**Conclusion:**

Our data from a German level 1 trauma centre proof that abdominal surgeons are most frequently required for the treatment of trauma patients with hypotension on arrival among all surgical disciplines (> thoracic surgery > vascular surgery > neurosurgery). Therefore, surgeons from these specialties must be available without delay to provide optimal trauma care.

## Background

Severe trauma is the most common cause of death in people < 45 years of age in the Western world [1] and is mostly related to traffic accidents in Europe and Germany [2]. In total, 28.580 patients with severe trauma were transferred to trauma units in Germany in 2021, causing a mortality of 12.5% [3].

The distribution of injured body regions with Abbreviated Injury Scale (AIS) grades and trauma mechanisms is published annually by the German Society for Trauma Surgery [3], with a proportion of blunt trauma > 95% within the last ten years.

Among 88.372 trauma patients from 2019 to 2021, injuries of the extremities (e.g., fractures; arms: 29.1%; legs: 23.1%) were most common, followed by cranial (e.g., traumatic brain injury, subdural and epidural hematoma, 45.5%), thoracic (e.g., rib fractures with and without pneumothorax, 45.2%), pelvic 15.4% and spine injuries 29.6%. Abdominal injuries are proportionally less represented (13.9%) [3].

In Germany, 23.9% of all trauma patients are transferred directly from the trauma unit to the operating theatre for an *immediate* surgical procedure (definition see below), with a mean time from admission to surgery of 77.7 min.

Only 7.7% of all trauma patients present with shock (defined as systolic blood pressure ≤ 90 mmHg on arrival), with an increased mortality rate of 32.2% [3]. A correlation between hypotension on admission and mortality in trauma patients has been previously demonstrated [4, 5]. Hypotension is an indicator for shock and blood loss, leading to hypovolemia and low cardiac output [6] and further causes trauma-induced coagulopathy [7]. In addition, it induces anaerobic metabolism with acidosis as a sign of tissue hypoxia as well as hypothermia [8, 9]. These combined physiological disorders are referred to as lethal trauma triad and must be treated immediately [7, 10].

The damage control surgery (DCS) concept was implemented by Rotondo and Schwab in 1993 [11] with focus on abbreviated surgical procedures in patients with life-threatening penetrating abdominal injuries and deranged physiological conditions (lethal trauma triad) [10]. DCS focuses on quick surgical interventions to achieve control of bleeding and contamination, thereby reducing the “second hit” of harmful, prolonged surgical procedures [12]. The physiological status of trauma patients will be restored in the intensive care unit (ICU) [13], and only thereafter further surgeries are performed to increase the chance of survival.

In many countries, such unstable trauma patients requiring immediate surgery are usually treated by specialized trauma surgeons who are capable of controlling abdominal and/or thoracic bleeding, and who also have emergency vascular surgery skills.

A trauma surgeon of this type is not established as a formal specialty in Germany. The surgical training regulations of recent years have led to ever earlier specialization [14], so that interdisciplinary surgical emergency care by a single person is no longer possible in the foreseeable future. Essential skills of an ideal trauma surgeon and possible ways of training under the increasing specialization of the present were discussed repeatedly [15–20].

Due to the high absolute number of extremity injuries and fractures associated with (blunt) trauma, it is not surprising that surgical treatment of these patients is most frequently performed by orthopaedic surgeons. However, surgical fracture treatment can in most cases be classified as *urgent*, but not as *immediate* interventions.

For treatment of patients with *immediate* need for surgery in European and specifically German trauma centres it is not evident (1) which surgical disciplines are most frequently required, (2) how often damage control protocols are used, and whether both these factors depend on or change with “hypotension on admission”.

### Aim

Thus, the aim of the study is to evaluate (1) the most common needed surgical disciplines for treatment, (2) the usage of damage control protocols, and (3) outcome of trauma patients with immediate surgery, and to analyse a possible dependence on “hypotension on arrival” (patients with shock).

## Methods

### Study design

This is a retrospective analysis of trauma patients with subsequent immediate surgery who were admitted to the German Armed Forces Hospital Ulm between January 2017 and September 2022. We are a certified level 1 trauma centre with a helicopter emergency medical service, and trauma treatment ranges from simple fractures to complex life-threatening injuries of all kinds. The following surgical disciplines are present in our hospital: abdominal surgery, thoracic surgery, orthopaedic surgery, vascular surgery, neurosurgery and maxillofacial surgery.

Prior to the study, it was approved by the ethics commission of the University of Ulm, Germany (approval number: 380/22).

### Data collection

Data were taken from the digital treatment documentation. Only patients with trauma and subsequent *immediate* surgeries were included, whereas patients with procedures classified as *urgent* interventions were excluded, according to the definition of the “*National Confidential Enquiry into Patient Outcome and Death (NCEPOD)*” [21]:

- *immediate* = immediate life, limb or organ saving intervention, normally within minutes of decision to operate.
- *urgent* = intervention for acute onset or clinical deterioration of potentially life- or limb threatening conditions, normally within hours of decision to operate.
- *expedited* = required early treatment where the condition is not an immediate threat to life or organ, normally within days of decision to operate.
- *elective* = intervention planned or booked in advance of routine admission to hospital.

Patients with SBP > 90 mmHg on admission to the trauma unit were defined as normotensive (group 1, no-shock), and patients with SBP < 90 mmHg were defined as hypotensive (group 2, shock), in accordance with the literature [22–26].

Trauma patients from all surgical disciplines were included in the analysis. Injuries leading to immediate surgery were collected with their corresponding surgical procedures and the leading surgical discipline.

Furthermore, patient characteristics such as laboratory values defining the lethal triad (pH, partial thromboplastin time and body temperature), SBP on admission, Injury Severity Score (ISS), prehospital intubation, the presence of free abdominal fluid during focused assessment with sonography in trauma (FAST) or CT, were evaluated. Procedural parameters such as usage of damage control protocols, timeframes, as well as outcome parameters (e.g. mortality and complications) were analysed. Surgical complications were graded using the Clavien‒Dindo classification [27].

Usage of damage control protocol was defined as any surgery with abbreviated surgical procedures, based on clearly documentation in the surgical reports, or in any cases when a temporary abdominal closure was performed.

### Statistical analysis

Data were anonymized and listed in a Microsoft Excel® table (Version 16.0, Microsoft, Redmond, Washington, United States of America). Descriptive statistics were used to show the demographic data, patient characteristics and the most involved surgical disciplines as well as the most common injuries and performed procedures.

Metrical data were examined for the level of significance between groups 1 and 2 using the Mann‒Whitney U test. Categorical data were investigated by Fisher’s exact test. Statistical analysis was performed using SPSS statistics® Version 26 by IBM (Armonk, New York, United States of America).

## Results

**Fig. 1:**
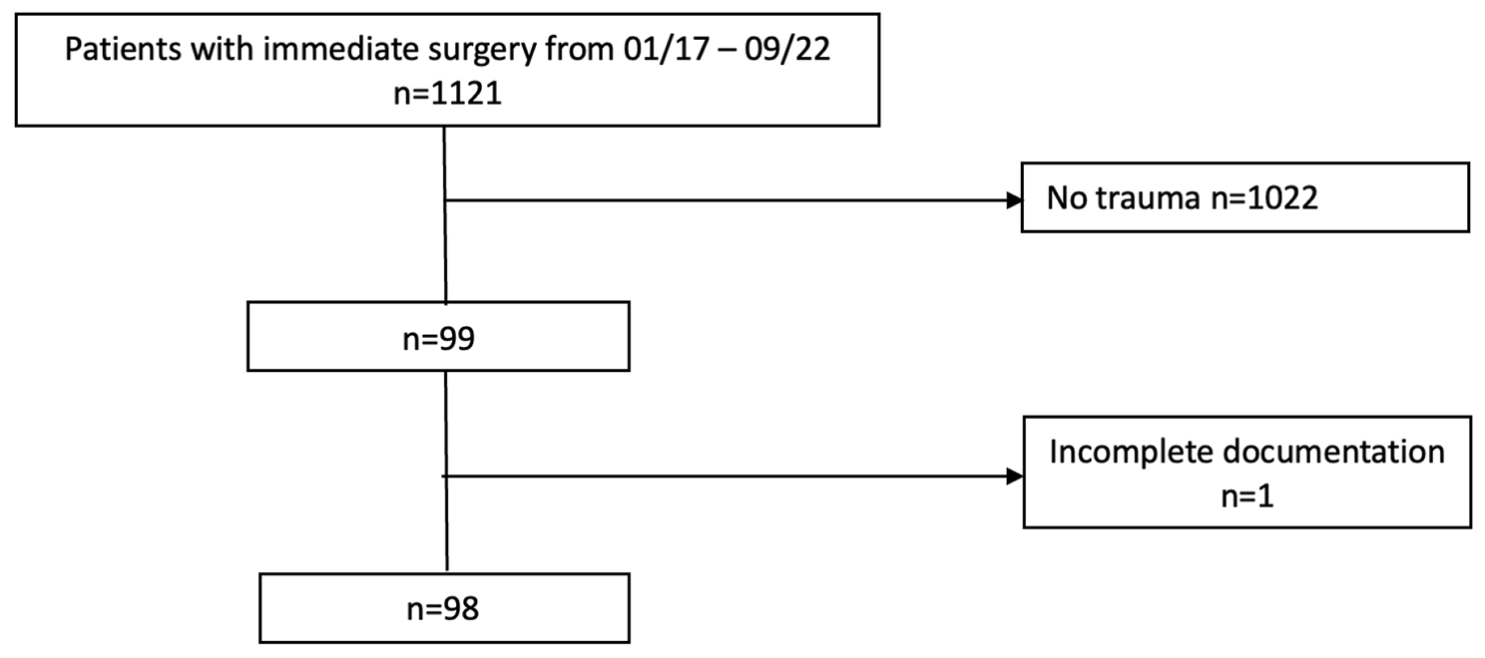
Flowchart of patient selection for final analysis

A total of 1.121 immediate surgeries in the study period were screened. Among these, 1.022 had to be excluded because surgery was not related to trauma but other conditions, and one patient had to be excluded due to an incomplete digital documentation. Finally, 98 trauma patients with immediate surgery were included in our study (Fig. 1).

Sixty-one (62%) patients were normotensive (group 1, SBP > 90 mmHg), and 37 (38%) were hypotensive (group 2, shock, SBP < 90 mmHg), on admission to the trauma unit.

Seventy (71%) patients were male, mean age was 44.8 years, mean ISS was 27.3 (ISS group 1: 24.6 vs. group 2: 32.8; *p* = 0.003), and 89 (90%) trauma mechanisms were blunt.

There were significant differences between group 1 and 2 in regard to pH < 7 (acidosis; group 1: 1.6 vs. group 2: 16.2%; *p* = 0.007), pTT > 60 s (coagulopathy; group 1: 6.5 vs. group 2: 21.6%; *p* = 0.027), preclinical intubation (group 1: 57.3 vs. group 2: 81.0%; *p* = 0.016), mass transfusion > 10 pRBC (group 1: 1.6 vs. group 2: 27.0%; *p* < 0.001), and free-abdominal fluid (group 1: 42.6 vs. group 2: 75.6%; *p* = 0.001) (Table 1).

**Table 1:** Demographic and on arrival measured values of included trauma patients with immediate surgery

	All patients [*n* = 98]	Systolic BP > 90 mmHg [*n* = 61]	Systolic BP < 90 mmHg [*n* = 37]	*p*-value
Age [years]	44.8	45.7	43.0	0.565
Male gender; n [%]	70 [71]	45 [73.7]	25 [67.5]	0.510
penetrating trauma; n [%]	9 [9.2]	5 [8.1]	4 [10.8]	0.664
ISS	27.3	24.6	32.8	**0.003**
Body temperature, < 35.0° C; n [%]	19 [19.4]	9 [14.7]	10 [27.0]	0.136
pH < 7.0; n [%]	7 [7.1]	1 [1.6]	6 [16.2]	**0.007**
pTT > 60 s; n [%]	12 [12.2]	4 [6.5]	8 [21.6]	**0.027**
Preclinical intubation; n [%]	65 [66.3]	35 [57.3]	30 [81.0]	**0.016**
Mass transfusion (> 10 pRBC); n [%]	11 [11.2]	1 [1.6]	10 [27.0]	**< 0.001**
Free abdominal fluid; n [%]	54 [55.1]	26 [42.6]	28 [75.6]	**0.001**

BP = blood pressure, C = celsius, ISS = injury severity score, PTT = partial thromboplastin time, pH = potentia hydrogenii, pRBC = packed red blood cell units

**Fig. 2:**
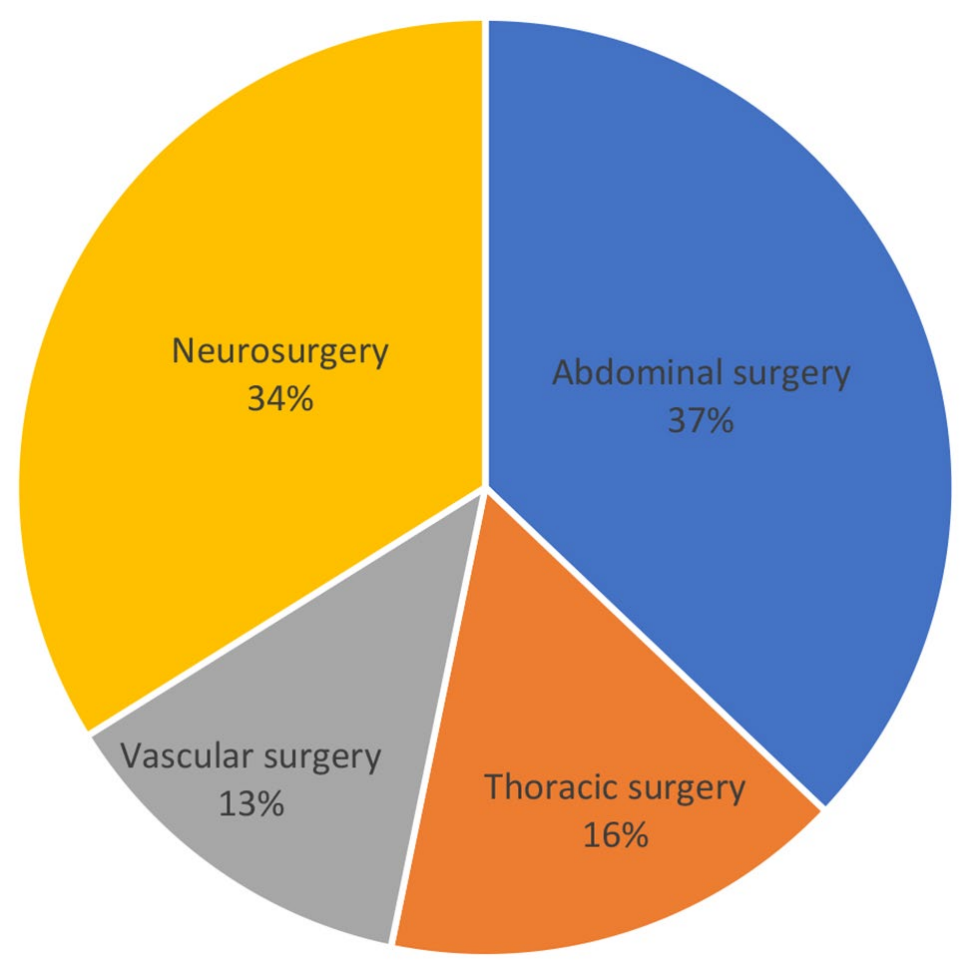
Percentages of performed surgeries by discipline on patients without hypotension on arrival (SBP > 90 mmHg)

**Fig. 3:**
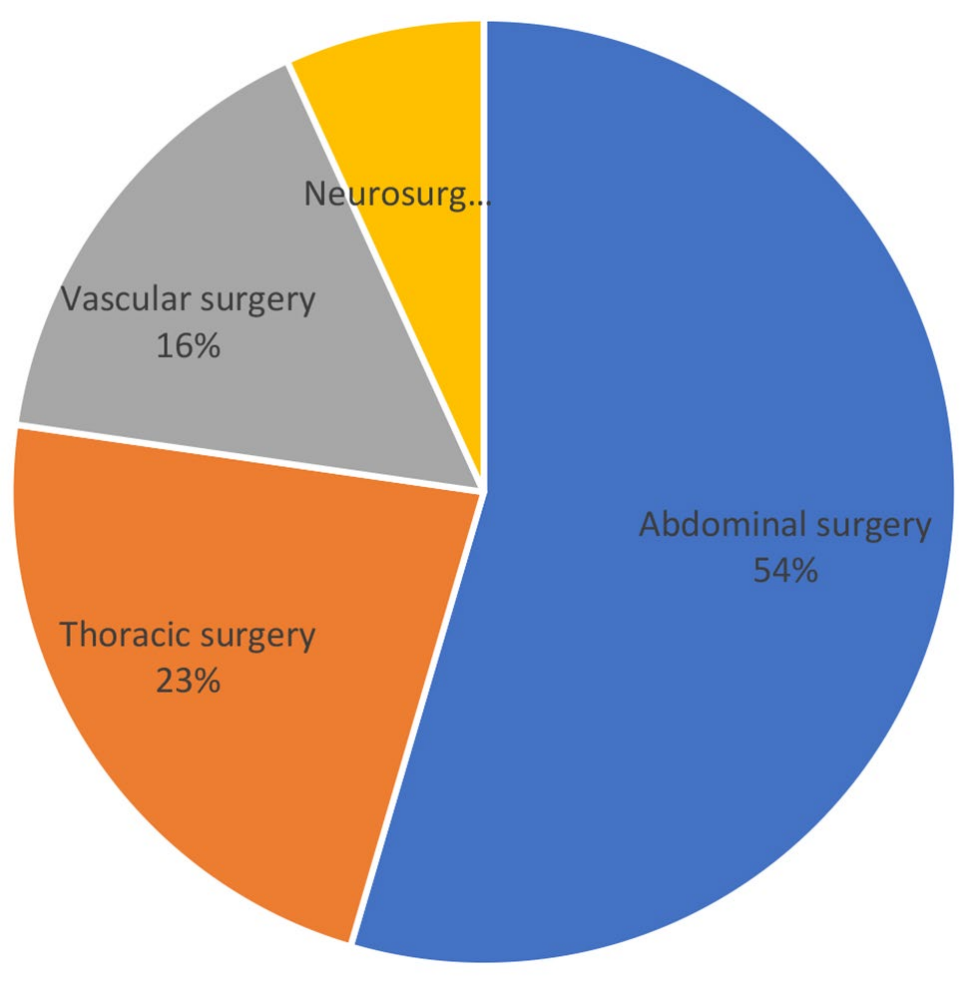
Percentages of performed surgeries by discipline on patients with hypotension on arrival (SBP < 90 mmHg)

The most common required surgical discipline for immediate surgical procedures was abdominal surgery for patients of both groups (all patients: 43.9%), with significant increase in patients with shock (abdominal surgery; group 1: 37.1 vs. group 2: 54.5%; *p* = 0.009) (Figs. 2 and 3). In contrast, the need of neurosurgery (all patients: 22.4%) decreased significantly with hypotension on admission (neurosurgery: group 1: 33.8 vs. group 2: 6.8%; *p* = 0.003). Other needed surgical disciplines for immediate interventions were thoracic (all patients: 18.6%) and vascular surgery (all patients: 14.9%), each without significant changes related to hypotension on admission (Table 2).

**Table 2:** Procedural and outcome parameters of included trauma patients with immediate surgery

	All patients [*n* = 98]	Systolic BP > 90 mmHg [*n* = 61]	Systolic BP < 90 mmHg [*n* = 37]	*p*-value
Time to CT [min]	23.1	17.8	28.3	0.104
Time to surgery [min]	99	112.1	82.6	**0.016**
Mortality; n [%]	14 [14.2]	5 [5.5]	9 [24.3]	**0.027**
Clavien-Dindo ≥ 3; n [%]	59 [60.2]	33 [54.1]	26 [70.3]	0.113
Length of ICU stay [days]	11.3	13	10.7	0.904
Length of hospital stay [days]	28.7	26.5	31.5	0.905
Invasive ventilation [days]	8.0	6.5	9.9	0.322
DCS protocol; n [%]	64 [65.3]	36 [59.0]	28 [75.6]	**0.019**
Total amount of surgeries	5.6	4.1	7.3	0.124
Emergency surgical procedures	107	62	44	
Abdominal surgery procedure; n [%]	47 [43.9]	23 [37.1]	24 [54.5]	**0.009**
Thoracic surgery procedure; n [%]	20 [18.6]	10 [16.1]	10 [22.7]	0.205
Vascular surgery procedure; n [%]	16 [14.9]	8 [12.9]	7 [15.9]	0.439
Neurosurgery procedure; n [%]	24 [22.4]	21 [33.8]	3 [6.8]	**0.003**

BP = blood pressure, CT = computed tomography, DCS = damage control surgery, ICU = intensive care unit, min = minutes

The usage of damage control protocols was 65% for all patients, with significant increase in patients with shock (damage control; group 1: 59.0 vs. group 2: 75.6%; *p* = 0.019). Time to surgery was significantly reduced in patients with hypotension on admission (group 1: 112.1 vs. group 2: 82.6 min; *p* = 0.016), and mortality was significant higher in the same group (group 1: 5.5 vs. group 2: 24.3%; *p* = 0.027). For all other outcome variables (complications according to Clavien-Dindo, length of ICU stay, days of invasive ventilation, length of hospital stay) no significant differences were observed (Table 2).

One hundred and seven surgical procedures were documented in 98 included trauma patients with immediate surgery. Among these, an explorative laparotomy was performed in 43.9% (*n* = 47), which was the most common procedure of all (associated abdominal interventions are shown in Table 3). There was no negative laparotomy (futile laparotomy without an intervention). Craniotomy was performed in 20.5% (*n* = 22), thoracotomy in 18.7% (*n* = 20) and arterial reconstructions in 8.4% (*n* = 9) (Table 3).

**Table 3:** Performed surgical procedures (more than one surgical procedure can be performed on one patient)

Performed surgical procedures (*n* = 107)	n [%]
**Abdominal surgery**	
Explorative Laparotomy	47 [43.9]
- Liver packing	15 [14.0]
- Splenectomy	13 [12.1]
- Temporary abdominal closure	13 [12.1]
- Bowel suture or resection	13 [12.1]
- Mesenteric suture	8 [7.5]
- Haemorrhage control spleen	8 [7.5]
- Other	8 [7.5]
**Neurosurgery**	24 [22.4]
Craniotomy	22 [20.5]
- with ICP probe	10 [9.3]
Osteosynthesis in paraplegia	2 [1.9]
**Thoracic Surgery**	20 [18.7]
Thoracotomy	20 [18.7]
- Relieve of haemothorax	15 [14.0]
- Pericardiotomy	3 [2.8]
- Other	3 [2.8]
**Vascular surgery**	19 [17.8]
- Arterial reconstruction	9 [8.4]
- Ligature	4 [3.7]
- Stenting	4 [3.7]
- Other	3 [2.8]

ICP = intracranial pressure

## Discussion

The primary purpose of our study was to evaluate the most common required surgical disciplines in trauma patients with indication for immediate surgery, and how this depends on hypotension on admission, in a German level 1 trauma centre.

Secondly, we wanted to assess the usage of damage control protocols in these selected trauma patients.

Thirdly, our aim was to analyse outcome (mortality) and possible differences between patients with no-shock (SBP > 90 mmHg) and shock (SBP < 90 mmHg) on admission.

We were able to show that abdominal surgery is the most common required discipline in patients with indication for immediate surgery, especially for haemorrhage in patients with shock. Other frequently needed surgical disciplines are neurosurgery (decreasing proportion in patients with hypotension on admission), thoracic surgery and vascular surgery.

To our knowledge, this is the first study with special focus on this topic. Our results, despite being obtained in a single centre with a relatively small patient cohort, may be relevant to optimize surgical staffing of level 1 trauma centres, particularly in the context of early subspecialisation in surgery. Since Germany and many other European countries do not have a trauma surgeon as a formal specialty, the treatment of severely injured patients is only possible as an interdisciplinary surgical approach. So which surgical disciplines do we really need for immediate interventions and potentially saving lives of those patients? And how is this affected by hypotension on admission as an indicator for shock? With this study, we presented an analysis to answer these important questions.

Abdominal, thoracic and vascular surgeons in Germany are mainly focused on elective and oncologic surgery. Based on the results of our study, the following implications arise: since these surgical disciplines most frequently perform immediate surgeries in trauma patients, they are encouraged to focus intensively on trauma, both theoretically and practically.

However, severe abdominal and thoracic trauma is relatively rare in absolute numbers in Germany. Thus, surgical skills from elective and oncologic surgery should be supplemented by trauma course formats to train specific procedures needed for trauma surgery.

In our cohort of trauma patients with immediate surgical interventions, the usage of damage control protocols was as high as 59%, even in patients with SBP > 90 mmHg on admission, and partially without any other triggers for damage control surgery (acidosis, coagulopathy, hypothermia). One possible reason for the very liberal usage may be that the surgical staff consists exclusively of military surgeons - and damage control principles are well known and trained with them. However, it was shown that overutilization of damage control surgery [28] and application of temporary abdominal closure [29] in patients without clear indications may be even harmful.

It should be noted that hypotension on admission is only a single parameter indicating shock in trauma patients. We did neither discriminate between volume responders and non-responders, nor did we take the use of catecholamines into account. However, hypotension on admission appears to be a parameter of high value in trauma patients, especially for rapid initial assessment of shock– as systolic blood pressure is very quick and easy to measure.

In our patients, hypotension on admission was associated with an increased mortality. Of note, there were further significant differences between the two groups, e.g., acidosis, coagulopathy and mass transfusion, each with a possible independent impact on mortality, as demonstrated before [8, 30].

Prolonged time to surgery may also influence mortality independently, especially in patients with shock. In stable trauma patients without shock, Harmsen et al. found no correlation between the on-site and prehospital time and the mortality rate [31].

Our study has several limitations. First, its retrospective nature has all known flaws and risks of bias. Secondly, the relatively small number of patients must be taken into account when the results are interpreted. For example, *p* values may not indicate differences that might have been found for a larger study cohort.

However, we focused exclusively on a very relevant subgroup of trauma patients with subsequent immediate surgery (with and without shock), and these patients are not very numerous, even in level 1 trauma centres.

Further studies are required to obtain more reliable results using multicentre data and a larger number of patients.

## Conclusion

Abdominal surgery is the most frequently required surgical discipline for trauma patients with subsequent immediate interventions, and even more so for patients with hypotension and shock on admission. Other disciplines frequently involved are neurosurgery, thoracic and vascular surgery.

Level 1 trauma centres must therefore provide a high level of trauma expertise in these surgical disciplines to treat patients with severe trauma appropriately.

Early surgical specialization will inevitably lead to higher staffing requirements for the treatment of trauma patients.

## Data Availability

The datasets generated and/or analyzed during this study can be obtained from the corresponding author on reasonable request.

## Abbreviations

AIS: Abbreviated injury scale
CT: Computer tomography
DCS: Damage control surgery
FAST: Focused assessment with sonography in trauma
ICU: Intensive care unit
ISS: Injury severity score
pTT: Partial thromboplastin time
pRBC: Packed red blood cells
SBP: Systolic blood pressure
